# The effects of music intervention on pediatric burn patients during treatment: a systematic review and meta-analysis

**DOI:** 10.3389/fneur.2025.1545611

**Published:** 2025-08-19

**Authors:** Bin Chen, Riti Qiu, Hua Huang

**Affiliations:** ^1^West China Second University Hospital, Sichuan University, Chengdu, China; ^2^Key Laboratory of Birth Defects and Related Diseases of Women and Children, Sichuan University, Chengdu, China; ^3^Music College of Sichuan Normal University, Chengdu, China; ^4^Longquanyi District of Chengdu Maernal and Child Health Care Hospital, Chengdu, China

**Keywords:** burn pediatric, music interventions, pain, anxiety, meta-analysis

## Abstract

**Background:**

Burns rank among the foremost causes of accidental death and injury in children and pediatric patients, and their treatment poses significant challenges. Music intervention has demonstrated considerable potential in alleviating pain and anxiety in pediatric burn patients during treatment. The present meta-analysis was designed to assess the impact of music intervention on the treatment of pediatric burn patients.

**Methods:**

We conducted a comprehensive search across multiple databases, including PubMed, Web of Science, Embase, and Cochrane Library, to identify studies that met inclusion criteria. Only RCTs published in English that evaluated the efficacy of music intervention in pediatric burn patients were considered eligible for inclusion. The quality assessment of the included studies was performed using the Cochrane risk-of-bias tool. This study was performed based on the Guidelines of Systematic Reporting of Examination presented in the PRISMA checklist. The search protocol has been registered at the PROSPERO International Prospective Register of Systematic Reviews.

**Results:**

Four RCTs met the inclusion criteria, involving 158 pediatric burn patients aged from 1 day to 20 years who received either live music therapy or recorded music. Overall, a statistically significant positive effect was observed on both pain [SMD = −0.43, 95% CI (−0.80, −0.07)] and anxiety [SMD = −0.66, 95% CI (−1.05, −0.28)]. However, the music intervention group did not show a statistically significant reduction in heart rate [SMD: 0.20, 95% CI (−0.49, 0.89)] or respiratory rate [SMD: −0.21, 95% CI (−0.90, 0.47)].

**Conclusion:**

Findings from this study indicates that Music intervention has a positive effect in alleviating pain and reducing anxiety in children and adolescents with burn injuries.

## 1 Introduction

Burns are a global public health problem, due to the immaturity of children’s physical and cognitive abilities, they are especially susceptible to burn injuries (1). Consequently, burns represent a leading cause of accidental death and injury in children, especially in undeveloped countries (2, 3). Burns are the third most common cause of accidental death in children aged 5 to 9 years (4), and approximately 84,000 children under the age of 14 years in the United States required medical treatment for burns in 2017 (5). The treatment of burn patients is very challenging because burn injuries are one of the most severe traumas (6), has physiological as well as psychological consequences (7, 8). The main sources of pain in burn patients are wound care procedures (9) and physical rehabilitation (10) that are repeated daily. In addition, anxiety associated with wound care procedures may exacerbate pain, increase pain intensity, or reduce the therapeutic effectiveness of medications (11, 12). The traditional approach is to administer analgesic drugs such as tramadol to relieve pain and anxiety caused by burns. Given that the administration of anesthetics and analgesics to severely burned patients can lead to hemodynamic instability, their safety is not assured (13). Furthermore, the use of sedatives and analgesics in pediatric patients, a particularly vulnerable population, necessitates stringent monitoring. Therefore, the treatment of pediatric burn patients must encompass a comprehensive approach to managing both pain and anxiety.

Music interventions have also been used to manage pain and anxiety in patients during medical procedures for many years. These interventions have typically been used during treatment and rehabilitation. Previous studies have reported the use of music as an intervention during dental surgeries (14), operations (15), chemotherapy (16) and even can reduce anxiety in ICU patients on mechanical ventilation (17, 18). Music as an intervention has wide applicability during burn treatment due to its non-pharmacological, non-invasive and easily accessible features. Music has the potential to influence the central nervous system and divert patients’ attention, facilitating their transition into a relaxed state and enhancing their psychological well-being (19). This conclusion is supported by the gate control theory, which posits that the activation of larger, faster-conducting sensory neurons can inhibit the transmission of pain signals (20). Additionally, music stimulates the release of endorphins and dopamine in the ventral striatum and prefrontal cortex, which directly reduce pain perception (参考文献). The advantage of music as a simple complementary therapy lies in that it is a non-invasive, low-cost and useful supplementary treatment method that does not require a high level of training (21). Previous meta-analyses have confirmed that music therapy can effectively alleviate patients’ pain and anxiety (10, 22, 23). Li et al. (10) conducted a meta-analysis on music interventions for burn patients but included both pediatric and adult populations (*n* = 17 RCTs, 804 patients) without focusing on pediatric-specific outcomes.

Our study’s differential contribution: (1) Exclusively focuses on pediatric burn patients (1 day to 20 years), addressing a critical gap in pediatric-specific evidence; (2) Includes more recent RCTs (2017–2022) not covered by Li et al.; (3) Examines age-related variability and specific outcomes (e.g., vital signs) in pediatric populations.

## 2 Methods

This study strictly adhered to the guidelines outlined in the Preferred Reporting Items for Systematic Reviews and Meta-Analyses (PRISMA) (24). The research plan was officially registered with the International Prospective Register of Systematic Reviews (PROSPERO; registration number CRD42024620982) on December 12, 2024. For statistical analysis we used Review Manager (RevMan 5.4) (The Nordic Cochrane Centre, Copenhagen, Denmark, 2020).

### 2.1 Search strategy

We performed an exhaustive search of all literature regarding the clinical application of music therapy on burn patients using the following databases: PubMed, EMBASE, Cochrane Library, and Web of Science, to identify English-language studies published from the inception until November 15, 2024. Both MESH terms and free text words describing ‘the use of music interventions (including music therapy and music medicine)’ and ‘the measurement of physical activity outcomes (including pain, anxiety, burn characteristics, dressing changes, wound care, debridement, and rehabilitation)’ were used in the search. The articles from both sets were subsequently combined using the Boolean ‘AND’ operator. We exclusively selected studies that involved children. Furthermore, the reference lists of the included articles and pertinent reviews were independently evaluated to identify additional studies that met the inclusion criteria of our study.

### 2.2 Eligibility criteria

The inclusion criteria were as follows: (1) study type: RCTs, regardless of blinding; (2) Study Participants: Pediatric burn patients aged 1 day to 20 years, undergoing various procedures such as dressing changes, debridement, range of motion exercises, rehabilitation training, and surgery, with no restrictions on sex, burn size, or burn depth; (3) Study Interventions: in the music group, participants received music interventions before, during, and/or after procedures. These interventions included both music therapy and music medicine, with music being either live or recorded and covering a wide range of styles. In contrast, the control group underwent procedures without any musical accompaniment; (4) study outcomes: The primary outcomes of interest are the changes in pain and anxiety levels. Secondary outcomes include basic vital signs, such as heart rate and respiratory rate; (5) sufficient data: availability of sufficient data for meta-analysis. Exclusion Criteria: (1) study designs other than RCTs, including quasi-randomized trials, non-randomized trials, observational studies, case reports, abstracts, or letters; (2) burn patients with hearing impairment, cognitive impairment, or communication disorders. (3) studies where the statistical data cannot be converted or applied; (4) publications in languages other than English; and (5) duplicate publications or original articles that are not accessible through multiple sources.

### 2.3 Data extraction

Data were carefully and independently extracted from all eligible studies by two investigators (R. Q, H. H) according to the inclusion criteria mentioned above using a prespecified Microsoft Excel spreadsheet. The extracted data included study characteristics (e.g., author name, year of publication, total body surface area (TBSA), sample size, and patient age), effect measurements (e.g., pain score, level of anxiety, and heart rate and respiratory rate), and quality indicators (e.g., adequate sequence generation, allocation concealment, and blinding). Disagreement was resolved by discussion or consulting with a third reviewer (B. C).

### 2.4 Quality assessment

The methodological quality of the studies was independently evaluated by two investigators (R. Q, H. H) according to the Cochrane Risk of Bias tool for RCTs (25). The assessment domains encompassed various aspects such as “randomization procedure,” “deviations from intended interventions,” “unavailable outcome data,” “outcome measurement,” “selection of reported outcomes,” and “overall bias.” Each domain was categorized as either “high risk, ““some concerns, “or “low risk. “Any differences were resolved by consulting with the corresponding author.

### 2.5 Statistical analysis

Statistical heterogeneity was determined using Q-test and the I^2^ statistic. For cases in which *p* ≤ 0.10 and I^2^ ≥ 50%, a random effects model was applied. Otherwise, a fixed effects model was used. The endpoints were SMDs and 95% CIs. Publication bias was assessed using Begg’s funnel plot and Egger’s test.

## 3 Results

### 3.1 Search results and study characteristics

**Figure 1:**
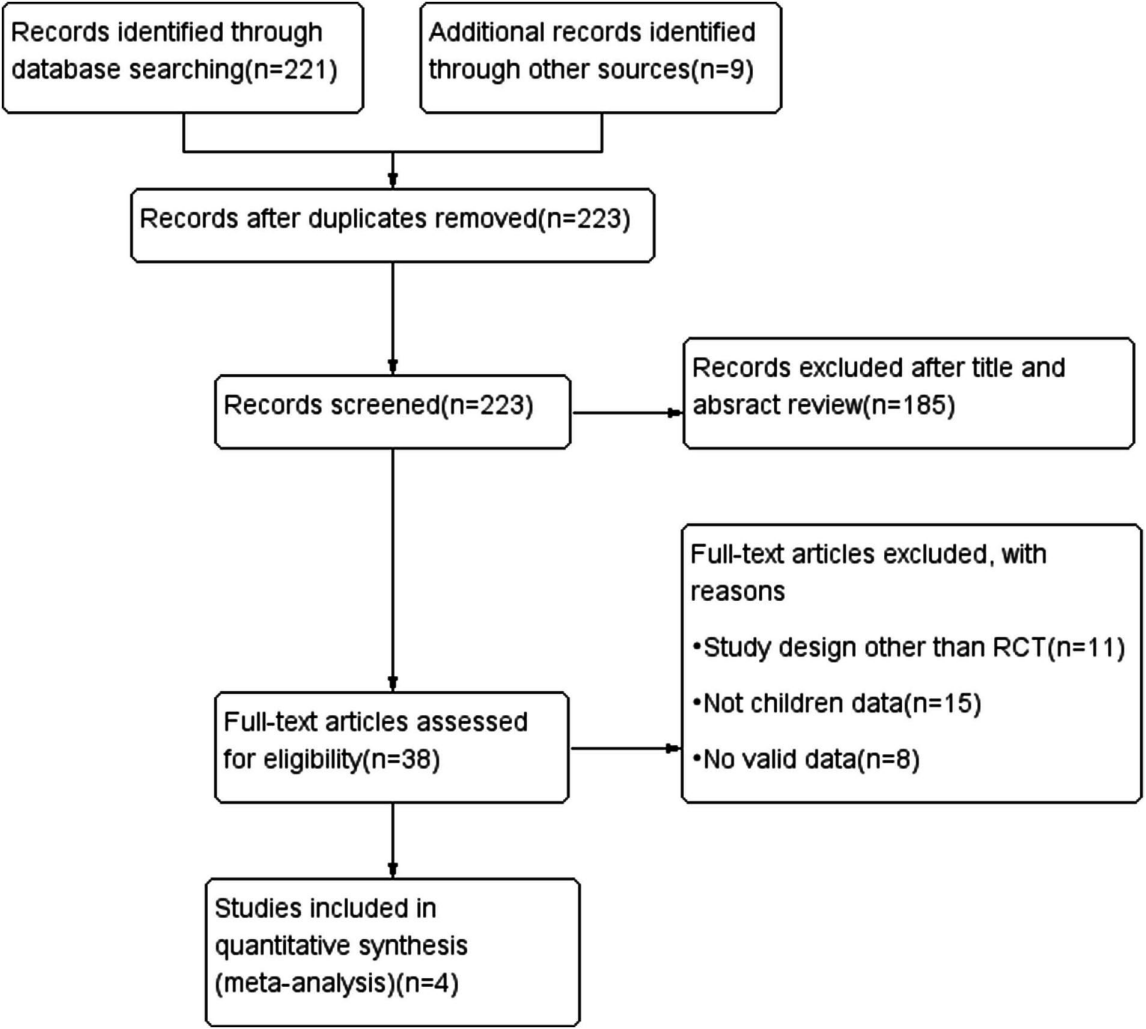
Flow chart of the literature search.

After performing an extensive electronic search combined with a manual search, 330 records were identified, resulting in an initial library of 223 references following the removal of 107 duplicates. 189 records were excluded on the basis of title or abstract. Thirty-eight full-text articles reviewed to determine its eligibility for inclusion and exclusion criteria. After an independent review of titles and abstracts, thirty-four records were excluded for failing to meet the inclusion criteria. A total of four RCTs (26–29) were included in the final review (Figure 1).

The following variables were extracted from the included studies: trial sample size, participant ages and genders, TBSA, procedures, music type, duration, measurement tools, control conditions, and outcomes. These data are summarized in Table 1. A total of 158 participants were enrolled, with 79 assigned to the music group and 79 to the control group. One study involving 20 patients did not provide gender information (26). The remaining three studies included a total of 138 patients, of whom 47.1% were male. Two studies did not provide TBSA information (26, 27). The procedures investigated included dressing changes (26, 27, 29) and rehabilitation training (28). Three studies compared with routine medical care (26, 28, 29), while one study compared it with verbal support interventions (27). One study used live music (27), two studies used record music (26, 29), and another study employed music with a powerful and steady beat, similar to the marching drum music of a military band, specifically composed for walking and typically performed with a military marching mood (28).

**Table 1:** Baseline characteristics and primary results of included trials.

Author, year, country	TBSA (%)	Age (year)	Sample M/F	Procedure	Music	Duration	Control	Scale for pain	Scale for anxiety	Outcome
Robb et al. (26)	N/A	8–20	T:10 C:10	Dressing change	Recorded music	30–50 min before and during procedure	CON		STAIC	①→③ ④
Whitehead-Pleaux et al. (27)	N/A	6–16	T:14/5 M/9F C:14/8 M/6F	Dressing change	Live music	During procedure	Verbal interaction	NAPI, WBFRS	FT	①→② ③ ④
Eid et al. (2021)	Less than 20%	10–15	T:15/8 M/7F C:15/6 M/9F	Rehabilitation training	Marching music	15 min during procedure	CON	VAS		①→
Shoghi et al. (29)	9 ∼ 35%	3–6	T:40/16 M/24F C:40/22 M/18F	Dressing change	Recorded music	Before and during dressing change	CON	VAS	OBSD-R	①→②

TBSA, total body surface area; NA, unavailable; T, experimental group; C, control group; M, male; F: female; CON, control group with routine care; STAIC, the state trait anxiety index for children; WBFRS, Wong/Baker faces rating scale; NAPI, the nursing assessment of pain index; VAS, visual analog scale; FT, the fear thermometer; OBSD-R, observational scale of behavioral distress-revised.

Outcome: ① = Pain ② = Anxiety ③ = hear rate ④ = Respiratory rate.

### 3.2 Outcome measurements

Pain intensity was assessed in three studies (27–29) using the following measurement tools: the Visual Analogue Scale (VAS) (28, 29), the Wong/ Baker Faces Rating Scale (WBFRS) (27), the Nursing Assessment of Pain Index (NAPI) (27). Three studies assessed anxiety descriptors (26, 27, 29) using the following measurement tools: State–Trait Anxiety Inventory forms (STAI) (26), the Fear Thermometer (FT) (27) and the Observational Scale of Behavioral Distress-Revised (OBSD-R) (29). Respiratory rate and heart rate were assessed in the two studies (26, 27) (Table 1).

### 3.3 Risk of bias

**Figure 2:**
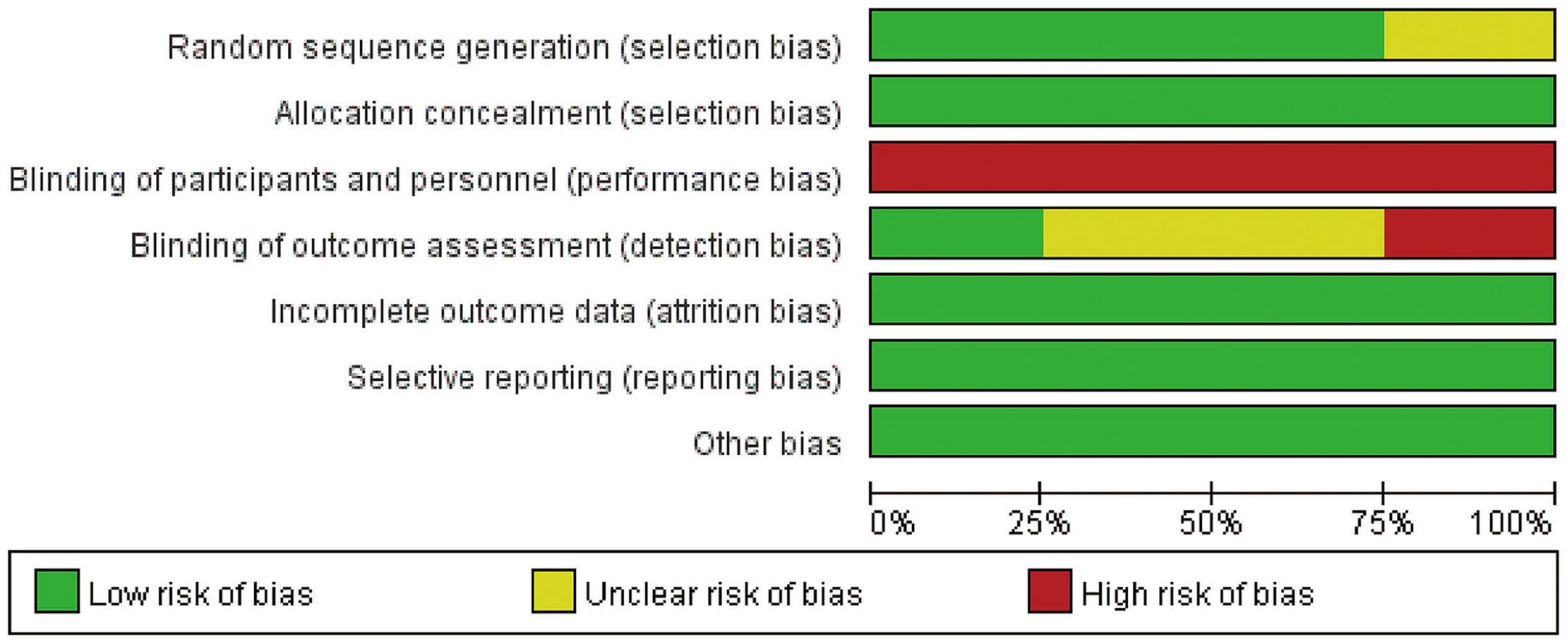
Risk of bias graph.

The risk of bias in the included studies is depicted in Figure 2. One study did not describe their exact methods of randomization (29). Due to the nature of music intervention, the double-blind evaluation criteria used in most studies are not realistic. Usually, only blinding of the evaluators can be considered. One study clearly stated that blinding of the evaluators was carried out (29). The four related literatures included in the study were of medium quality and were considered acceptable for inclusion.

### 3.4 Primary outcome

#### 3.4.1 Pain

**Figure 3:**
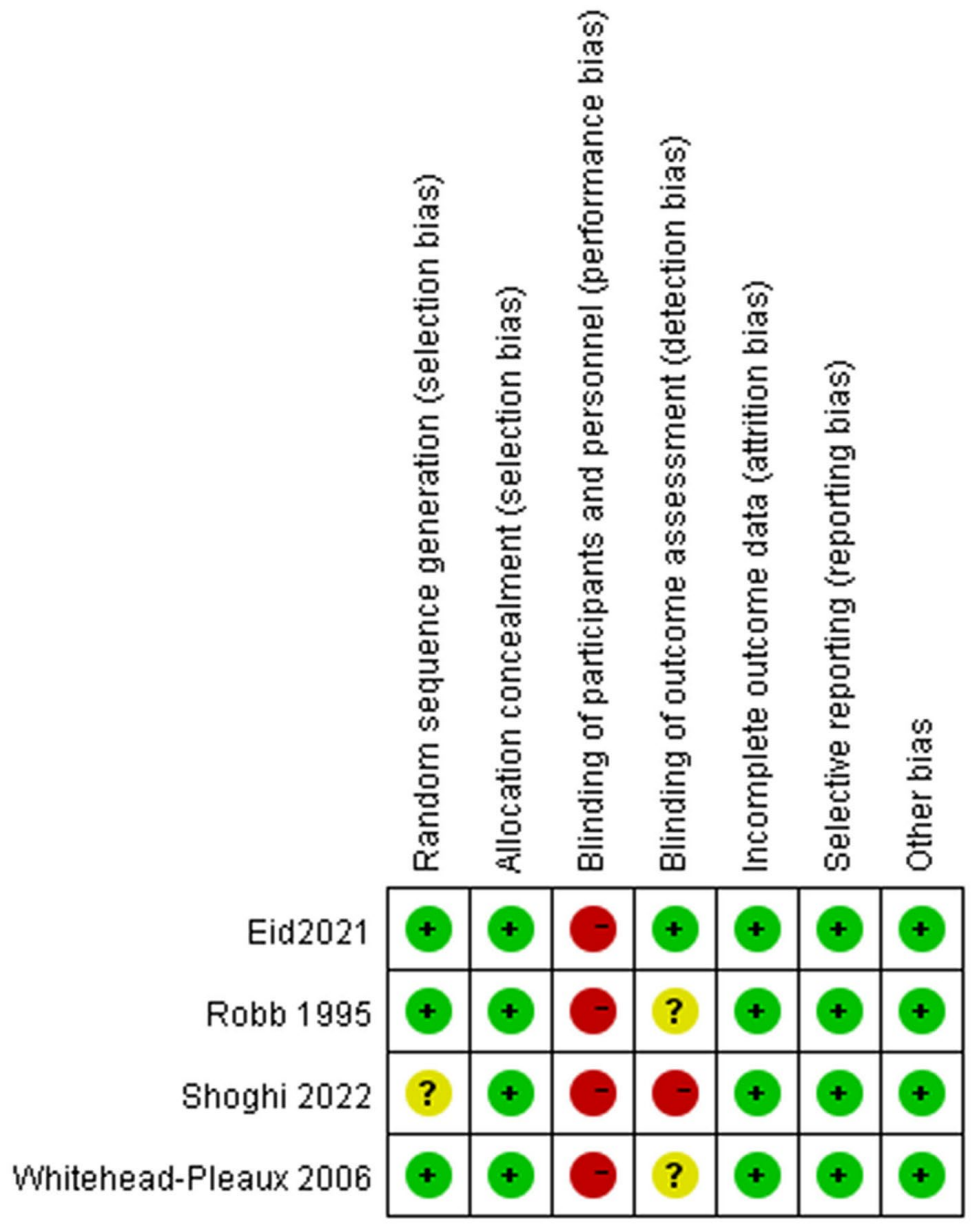
Risk of bias summary.

**Figure 4:**
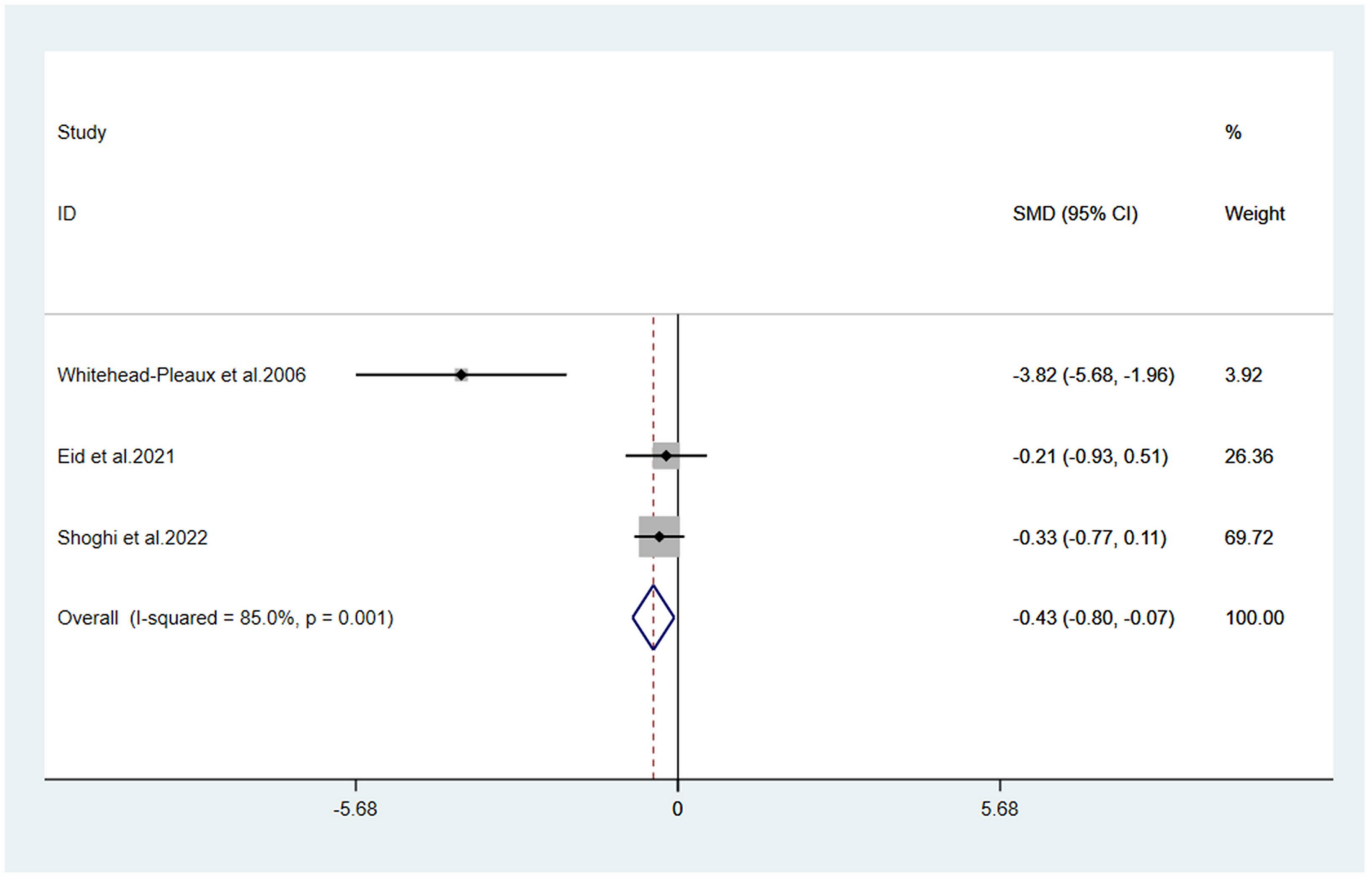
Forest plot of pain.

The meta-analysis of three trials with 138 burn patients for measures of pain intensity demonstrated significant heterogeneity (I^2^ = 81.6%, *P*<0.1). The pooled result from the random effects model demonstrated significant differences in pain scores between the music intervention group and the non-music intervention group [SMD = −0.43, 95% CI (−0.80, −0.07)] (26–29) (Figures 3, 4). Music intervention was found to reduce the pain experienced by burn patients during treatment procedures.

#### 3.4.2 Anxiety

**Figure 5:**
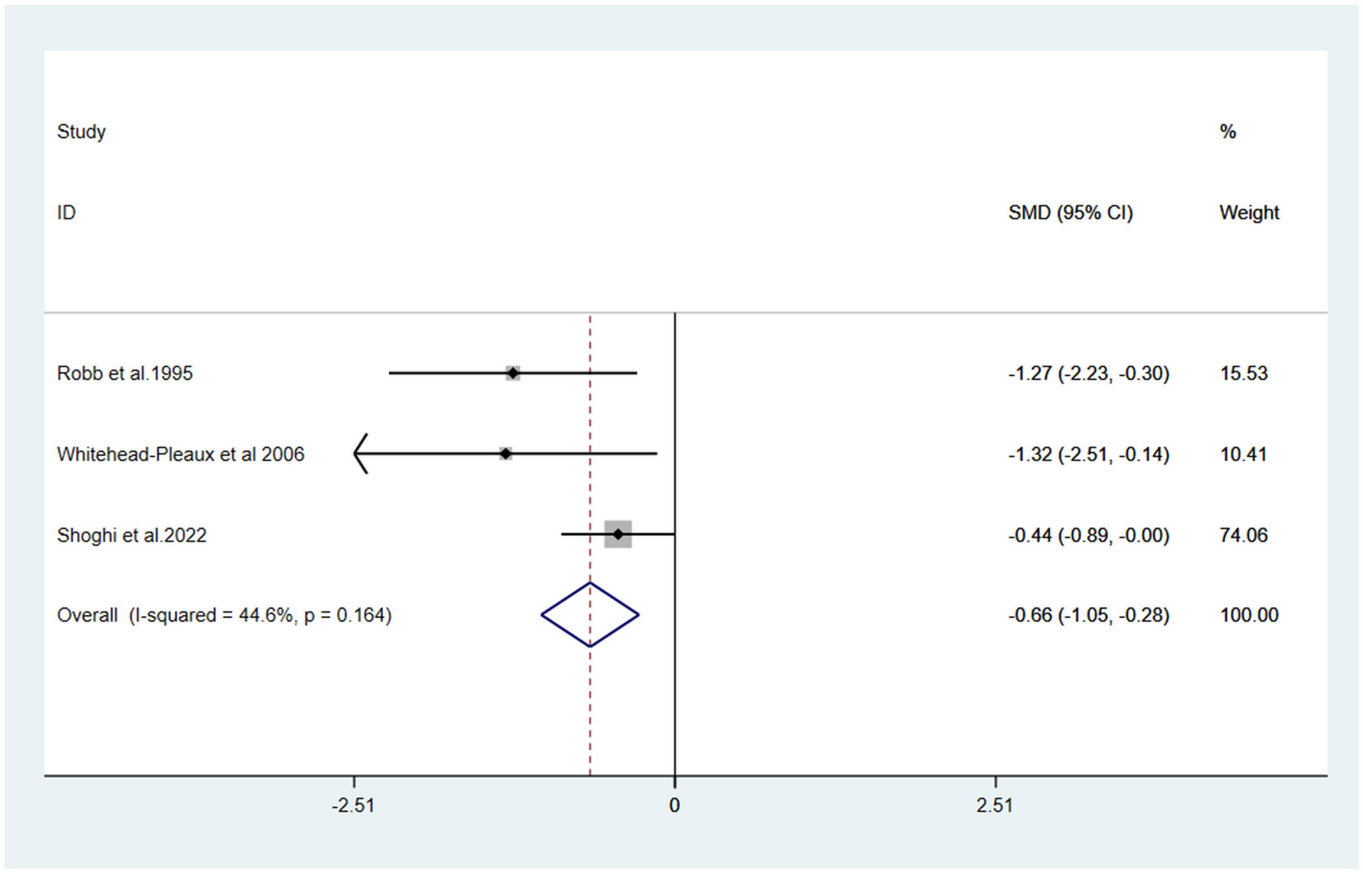
Forest plot of anxiety.

The included anxiety scores exhibited statistically insignificant heterogeneity (I^2^ = 44.6%, *P* > 0.1). The pooled result from the fixed effects model showed a statistically significant reduction in the anxiety levels of the burn patients [SMD = −0.66, 95% CI (−1.05, −0.28)] in the music intervention group compared to those in the non-music intervention group (26, 27, 29) (Figure 5).

### 3.5 Secondary outcome

**Figure 6:**
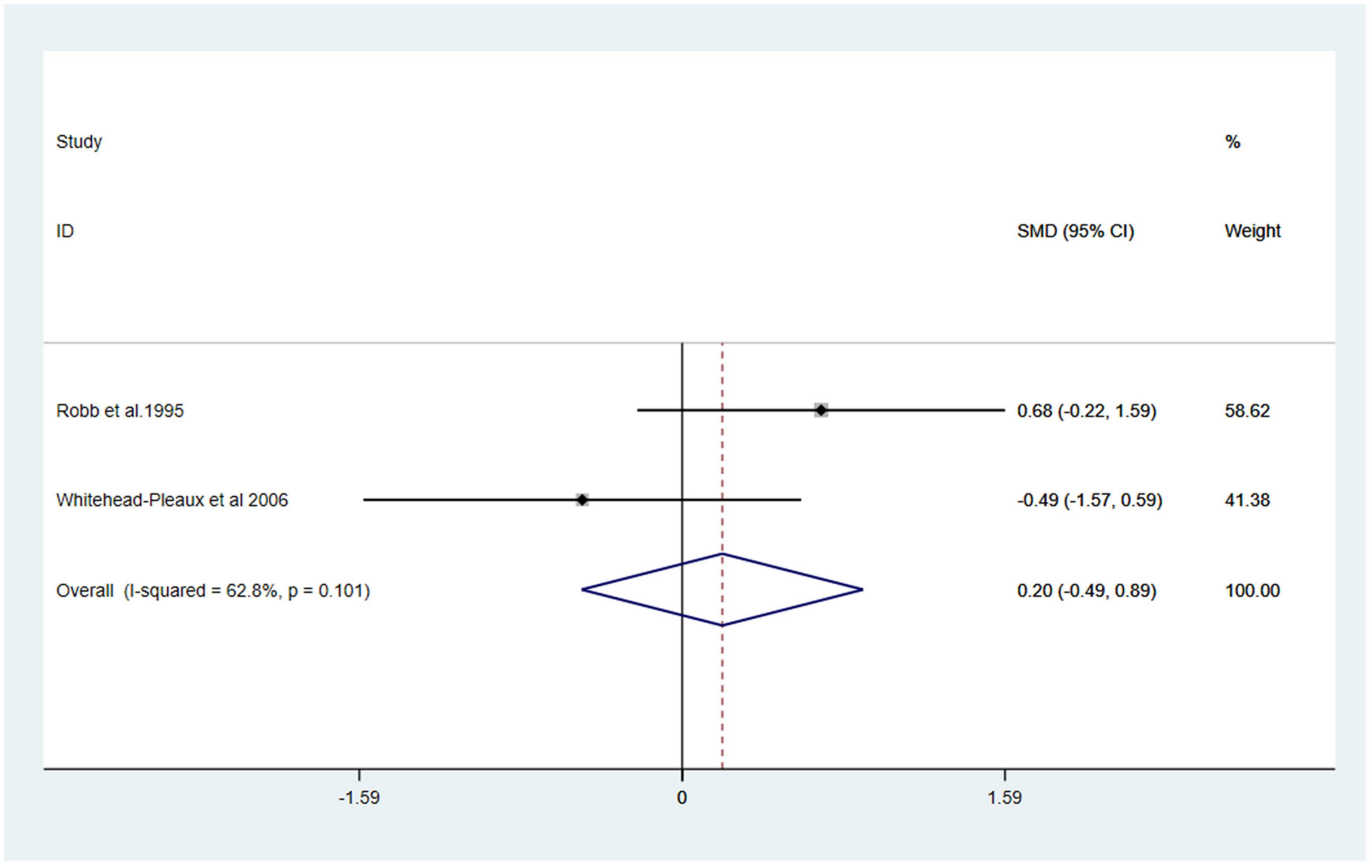
Forest plot of heart rate.

**Figure 7:**
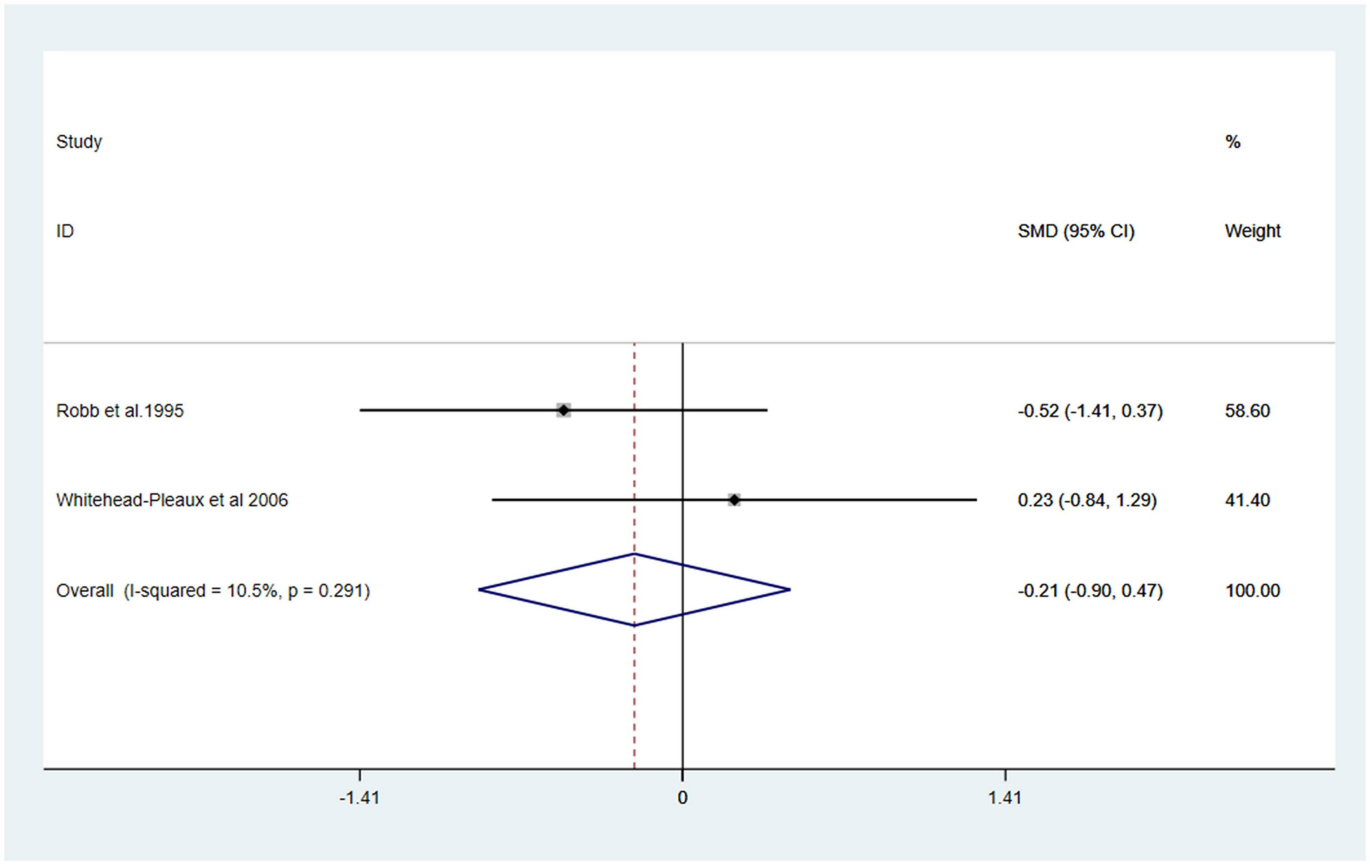
Forest plot of respiratory rate.

The effects of music intervention on heart rate and respiratory rate during burn treatment procedures were analyzed based on data from two studies included in the meta-analysis (26, 27). Compared with the usual care group, the music intervention group did not exhibit a statistically significant reduction in either heart rate [SMD: 0.20, 95% CI (−0.49, 0.89, *p* = 0.101) I^2^ = 62.8%] or respiratory rate [SMD: -0.21, 95% CI (−0.90, 0.47) *P* > 0.1, I^2^ = 10.5%] (Figures 6, 7).

## 4 Discussion

The objective of this systematic review is to evaluate the impact of music interventions on pediatric burn patients during treatment or rehabilitation. Our research findings indicate that music effectively reduces pain and anxiety in children and adolescents undergoing burn treatment. The meta-analysis of the four relevant studies consistently supports these conclusions. Regarding pain intensity, one study reported a significant reduction in pain in the intervention group compared to the control group, whereas the other two studies found no significant effect of music on pain relief. Concerning anxiety levels, two studies indicated a significant reduction in anxiety in the intervention group relative to the control group, while another study found no significant effect of music on anxiety. The diversity of anxiety measurement (STAI for state anxiety, Fear Thermometer for fear, OBSDR for behavioral distress) likely contributed to residual heterogeneity in anxiety outcomes (I^2^ = 44.6%). We note that despite these differences, all measures showed a consistent trend of reduced negative emotional states in the music group, suggesting a robust effect of music on alleviating negative affect broadly. When comparing pre- and post-intervention measures within the intervention group, all studies demonstrated a significant decrease in pain and anxiety. This discovery aligns with the findings of a previous meta-analysis. Li et al. (10) conducted a meta-analysis encompassing 17 RCTs involving 804 burn patients to assess the impact of music during treatments. Their results demonstrated a positive correlation between music interventions and pain alleviation, anxiety relief, and heart rate reduction in burn patients. Wu et al. (22) focused on the optimal timing and effects of music therapy in burn injury patients, revealing that music therapy has a significant impact on background pain and anxiety. Zhang et al. (23) performed a network meta-analysis to evaluate the impact of music therapy and virtual reality (VR) therapy on burn patients. Their findings indicate that both music therapy and VR are effective in reducing pain and anxiety, with music therapy showing a significant advantage in pain relief, while VR technology is the most effective intervention for anxiety reduction. They recommend music therapy for pain management and VR therapy for anxiety management. Our findings align with their conclusion that music reduces pain/anxiety but add specificity to pediatric populations. We note Zhang et al.’s finding that music is superior to VR for pain, which supports our focus on music as a feasible pediatric intervention.

Music is known to modulate pain and anxiety through multiple neural pathways: Consistent with the gate control theory (20), music activates larger sensory neurons (e.g., Aβ fibers) to inhibit pain signal transmission in the spinal dorsal horn (30). Additionally, music stimulates the release of endorphins and dopamine in the ventral striatum and prefrontal cortex, which directly reduce pain perception (31). Music intervention affects the amygdala (a key region for emotional processing) by reducing its hyperactivity, which is associated with anxiety (32). It also modulates the hypothalamic–pituitary–adrenal (HPA) axis, lowering cortisol levels (33).

For children, the process of severe burns is often terrifying and painful. They may need extra time to explain more procedures, and also need to rest when they become severely distressed or aggressive. If the intervention of music therapy can improve the compliance of pediatric patients, then the time spent repeatedly soothing patients, taking breaks due to severe pain, or dealing with patients who try to resist treatment will be much less. Greater compliance may help reduce the cost of medical procedures.

The forms of music intervention utilized in our research are diverse, and additional studies are required to identify the most effective practices for using music-based interventions to alleviate pain and anxiety. The impact of patient-selected music, the type of playback equipment (e.g., headphones, speakers, live performances), the volume of the music, and the duration of listening on pain and anxiety levels remains unclear. Some studies suggest that music may not be the primary factor influencing pain and anxiety scores but rather serves as a general auditory distraction. Suresh et al. (34) compared pain scores between participants exposed to music and those listening to audiobooks, demonstrating that audio therapy can be an effective adjunctive method to reduce post-surgical pain in children undergoing major surgeries. While Kim et al. (35) found no statistically significant differences in anxiety scores between children who heard their mothers singing (music) and those who heard their mothers speaking (non-music). We connect Suresh et al. and Kim et al. to discuss whether music acts via distraction (vs. specific auditory features), noting that our results support distraction as a potential mechanism (consistent with gate control theory). Further research is needed to investigate the comparative effectiveness of music versus other audio therapies.

The broad age range (covering neonates to adolescents) is a critical consideration, as developmental stages influence music perception, pain/anxiety expression, and response to intervention. Infants (1 day to 2 years) rely on physiological/behavioral cues (e.g., crying, heart rate) for pain/anxiety assessment, while older children/adolescents use self-report scales (e.g., VAS). Music preferences also vary (e.g., lullabies for infants vs. preferred genres for adolescents). Age-related differences may contribute to heterogeneity in pain outcomes (I^2^ = 81.6%). Subgroup analyses by age (e.g., <5 years vs. ≥5 years) were attempted but limited by small sample sizes in individual studies. Future research should stratify by age to clarify whether music interventions are more effective in specific developmental stages.

This systematic review and meta-analysis represented the first effort to assess the efficacy of music interventions for pediatric burn patients during treatment, based on RCTs. Nevertheless, the findings should be interpreted with caution due to several limitations inherent in the original studies. Firstly, among the four included studies, the risk of bias was generally moderate. The overall trial quality was compromised due to the absence of concealed allocation and blinded therapist assessment of outcomes. Secondly, the number of included RCTs was limited, and the sample sizes in most of these trials were relatively small. The I^2^ statistic (e.g., 81.6% for pain) may overestimate heterogeneity due to sparse data, and the Q-test may lack sensitivity to detect true heterogeneity. We emphasize that the significant pooled effects (pain: SMD = -0.43; anxiety: SMD = -0.66) should be interpreted with caution, and larger-scale RCTs are needed to validate the findings. Thirdly, there was significant heterogeneity across the studies in terms of patient populations, types of music interventions, and treatment modalities. Fourthly, Music may evoke negative emotions (e.g., nostalgia, trauma) if not patient-selected, especially in adolescents with prior negative associations with specific genres. No included studies reported such adverse events, possibly due to the use of neutral/soothing music. Future studies should monitor for adverse reactions and prioritize patient-selected music to mitigate risks.

## 5 Conclusion

In conclusion, our findings from four randomized controlled trials provide evidence that music intervention can serve as a valuable complementary strategy in the management of pediatric burn patients, helping to alleviate pain and reduce anxiety during treatment. Importantly, music intervention is not proposed as a replacement for standard pharmacological or clinical care—such as analgesics, anesthetics, or routine medical procedures—but rather as an adjunctive approach that enhances holistic management. By addressing pain and anxiety through a non-pharmacological, non-invasive means, music intervention contributes to a more comprehensive treatment framework, potentially improving patient comfort, compliance, and overall treatment experience without compromising the efficacy of standard care. Given the limitations of the current evidence—including moderate risk of bias in included studies, small sample sizes, and heterogeneity in intervention protocols—further high-quality research with larger cohorts is warranted. Future studies should explore optimal parameters of music intervention (e.g., type, duration, delivery method) and its long-term effects, to better define its role in supporting pediatric burn patients alongside established clinical practices. Ultimately, integrating music intervention into holistic care plans may offer a safe and accessible way to enhance the well-being of this vulnerable population during treatment.

## Data Availability

The datasets presented in this study can be found in online repositories. The names of the repository/repositories and accession number(s) can be found in the article/Supplementary material.

## References

[1] Klas KS Vlahos PG McCully MJ Piche DR Wang SC. School-based prevention program associated with increased short- and long-term retention of safety knowledge. J Burn Care Research. (2015) 36:387–93. doi: 10.1097/bcr.0000000000000151 25159554

[2] Yin S. Chemical and common burns in children. Clin Pediatr. (2017) 56:8S–12S. doi: 10.1177/0009922817706975 28420255

[3] Elrod J Schiestl CM Mohr C Landolt MA. Incidence, severity and pattern of burns in children and adolescents: an epidemiological study among immigrant and Swiss patients in Switzerland. Burns. (2019) 45:1231–41. doi: 10.1016/j.burns.2019.02.009, PMID: 31097353

[4] Romanowski KS Palmieri TL. Pediatric burn resuscitation: past, present, and future. Burns Trauma. (2017) 5. doi: 10.1186/s41038-017-0091-y PMC5582395 28879205

[5] Partain KP Fabia R Thakkar RK. Pediatric burn care: new techniques and outcomes. Current Opinion Pediatrics. (2020) 32:405–410. doi: 10.1097/mop.0000000000000902 32371842

[6] Fratianne RB Prensner JD Huston MJ Super DM Yowler CJ Standley JM. The effect of music-based imagery and musical alternate engagement on the burn debridement process. J Burn Care Rehab. (2001) 22:47–53. doi: 10.1097/00004630-200101000-00010 11227684

[7] Carrougher GJ Brych SB Pham TN Mandell SP Gibran NS. An Intervention Bundle to Facilitate Return to Work for Burn-Injured Workers: Report From a Burn Model System Investigation. J Burn Care Rehab. (2016) 38:e70–e78. doi: 10.1097/bcr.0000000000000410 28009697

[8] Shepherd L Reynolds DP Turner A O’Boyle CP Thompson AR. The role of psychological flexibility in appearance anxiety in people who have experienced a visible burn injury. Burns. (2018) 45:942–949. doi: 10.1016/j.burns.2018.11.015 30591252

[9] De Jong AE Middelkoop E Faber AW Van Loey NE. Non-pharmacological nursing interventions for procedural pain relief in adults with burns: a systematic literature review. Burns. (2007) 33:811–827. doi: 10.1016/j.burns.2007.01.005 17606326

[10] Li J Zhou L Wang Y. The effects of music intervention on burn patients during treatment procedures: a systematic review and meta-analysis of randomized controlled trials. BMC Complementary Alternative Medicine. (2017) 17. doi: 10.1186/s12906-017-1669-4 PMC5356403 28302117

[11] Miller AC Hickman LC Lemasters GK. A distraction technique for control of burn pain. J Burn Care Rehabil. (1992) 13:576–80. doi: 10.1097/00004630-199209000-00012, PMID: 1452593

[12] Richardson P Mustard L. The management of pain in the burns unit. Burns. (2009) 35:921–36. doi: 10.1016/j.burns.2009.03.003, PMID: 19505764

[13] Summer GJ Puntillo KA Miaskowski C Green PG Levine JD. Burn injury pain: the continuing challenge. J Pain. (2007) 8:533–48. doi: 10.1016/j.jpain.2007.02.426 17434800

[14] Mejía-Rubalcava C Alanís-Tavira J Mendieta-Zerón H Sánchez-Pérez L. Changes induced by music therapy to physiologic parameters in patients with dental anxiety. Complement Ther Clin Pract. (2015) 21:282–6. doi: 10.1016/j.ctcp.2015.10.005, PMID: 26573456

[15] Bradt J Dileo C Shim M. Music interventions for preoperative anxiety. Cochrane Database Syst Rev. (2013) 2013:CD006908. doi: 10.1002/14651858.CD006908.pub2, PMID: 23740695 PMC9758540

[16] Lesiuk T. The effect of mindfulness-based music therapy on attention and mood in women receiving adjuvant chemotherapy for breast Cancer: A pilot study. Oncol Nurs Forum. (2015) 42:276–82. doi: 10.1188/15.Onf.276-282, PMID: 25901379

[17] Chlan LL Weinert CR Heiderscheit A Tracy MF Skaar DJ Guttormson JL . Effects of patient-directed music intervention on anxiety and sedative exposure in critically ill patients receiving mechanical ventilatory support: a randomized clinical trial. JAMA. (2013) 309:2335–44. doi: 10.1001/jama.2013.5670, PMID: 23689789 PMC3683448

[18] Chlan L. Effectiveness of a music therapy intervention on relaxation and anxiety for patients receiving ventilatory assistance. Heart Lung. (1998) 27:169–76. doi: 10.1016/s0147-9563(98)90004-8, PMID: 9622403

[19] Nilsson U. The effect of music intervention in stress response to cardiac surgery in a randomized clinical trial. Heart Lung. (2009) 38:201–7. doi: 10.1016/j.hrtlng.2008.07.008, PMID: 19486788

[20] Melzack R Wall PD. Pain mechanisms: a new theory. Science (New York, NY). (1965) 150:971–9. doi: 10.1126/science.150.3699.971, PMID: 5320816

[21] Najafi Ghezeljeh T Mohades Ardebili F Rafii F Haghani H. The effects of music intervention on background pain and anxiety in burn patients: randomized controlled clinical trial. J Burn Care Research. (2016) 37:226–34. doi: 10.1097/bcr.0000000000000266, PMID: 26132048

[22] Wu T Chen K Chiu W Lee C Wang H Kang Y . Optimal timing and effect of music therapy in patients with burn injuries: systematic review and meta-analysis of randomized controlled trials. Burns. (2022) 48:1069–78. doi: 10.1016/j.burns.2021.07.016, PMID: 34426015

[23] Zhang W Sui X Zhang L Zhang L Yan H Song S. Effects of two non-drug interventions on pain and anxiety in the nursing process of burn patients: a literature review with meta-analysis. Front Rehab Sci. (2024) 5. doi: 10.3389/fresc.2024.1479833, PMID: 39534646 PMC11554659

[24] Moher D Liberati A Tetzlaff J Altman DG. Preferred reporting items for systematic reviews and meta-analyses: the PRISMA statement. PLoS Med. (2009) 6:e1000097. doi: 10.1371/journal.pmed.1000097, PMID: 19621072 PMC2707599

[25] Sterne J Savović J Page M Elbers R Blencowe N Boutron I . RoB 2: a revised tool for assessing risk of bias in randomised trials. BMJ. (2019):366l4898. doi: 10.1136/bmj.l4898 31462531

[26] Robb SL Nichols RJ Rutan RL Bishop BL Parker JC. The effects of music assisted relaxation on preoperative anxiety. J Music Therapy. (1995):322–1. doi: 10.1093/jmt/32.1.2

[27] Whitehead-Pleaux AM Baryza MJ Sheridan RL. The effects of music therapy on pediatric patients' pain and anxiety during donor site dressing change. J Music Ther. (2006) 43:136–153. doi: 10.1093/jmt/43.2.136 16897906

[28] Marwa ME Walid Kamal A Fatma Moustafa A Ali ZA. Effect of physical therapy rehabilitation program combined with music on children with lower limb burns: A twelve-week randomized controlled study. Burns. (2020) 47:5. doi: 10.1016/j.burns.2020.11.006 33288333

[29] Shoghi M Aghtaii MZ Kheradmand M. The effect of the active and passive distraction techniques on the burn children's pain intensity and anxiety during dressing changes. J Nurs Midwifery Sci. (2022) 9:9167–72.

[30] Edwards C Alder T Rose GJ. Auditory midbrain neurons that count. Nature Neuroscience. (2002) 5:934–6. doi: 10.4103/jnms.jnms_139_21 12219094

[31] Arnold CA Bagg MK Harvey AR. The psychophysiology of music-based interventions and the experience of pain. Front Psychol. (2024) 15. doi: 10.3389/fpsyg.2024.1361857 PMC11122921 38800683

[32] Haoyang N Susu D Yanru F Honggui L Jianhong L Xiang L . Effect of noise and music on neurotransmitters in the amygdala: the role auditory stimuli play in emotion regulation. Metabolites. (2023) 13. doi: 10.3390/metabo13080928 PMC10456833 37623873

[33] Sittler MC Worschech F Wilz G Fellgiebel A Wuttke-Linnemann A. Psychobiological mechanisms underlying the health-beneficial effects of music in people living with dementia: a systematic review of the literature. Physiol Behav. (2021) 233. doi: 10.1016/j.physbeh.2021.113338 33497696

[34] Suresh BSS De Oliveira GS Jr Suresh S. The effect of audio therapy to treat postoperative pain in children undergoing major surgery: a randomized controlled trial. Pediatric Surgery International. (2015) 31:197–201. doi: 10.1007/s00383-014-3649-9 25555856

[35] Kim SJ Oh YJ Kim KJ Kwak YL Na S. The effect of recorded maternal voice on perioperative anxiety and emergence in children. Anaesthesia Intensive Care. (2011) 38:1064–1069. doi: 10.1177/0310057x1003800617 21226439

