# Supplementary material for: The effects of music intervention on pediatric burn patients during treatment: a systematic review and meta-analysis
Source: Front Neurol. 2025 Aug 19;16:1545611. doi: 10.3389/fneur.2025.1545611 (PMC12402896; doi:10.3389/fneur.2025.1545611)
Supplement: Supplementary file 1 [file Table_1.docx]

**Table1** Baseline characteristics and primary results of included trials

| Author, year, country | TBSA (%) | Age  (year) | Sample M/F | Procedure | Music | Duration | Control | Scale for pain | Scale for Anxiety | Outcome |
| --- | --- | --- | --- | --- | --- | --- | --- | --- | --- | --- |
| Robb et al.1995 | N/A | 8-20 | T:10  C:10 | Dressing change | Recorded music | 30-50 min before and during procedure | CON |  | STAIC | 1. ③ ④ |
| Whitehead-Pleaux et al.2006 | N/A | 6–16 | T:14/5M/9F  C:14/8M/6F | Dressing change | Live music | During procedure | Verbal interaction | NAPI, WBFRS | FT | 1. ② *③*   ④ |
| Eid et al.2021 | Less than 20% | 10-15 | T:15/8M/7F C:15/6M/9F | Rehabilitation training | Marching music | 15 min during procedure | CON | VAS |  |  |
| Shoghi et al.2022 | 9∼35% | 3-6 | T:40/16M/24F C:40/22M/18F | Dressing change | Recorded music | Before and during dressing change | CON | VAS | OBSD-R | 1. ② |

Abbreviations: TBSA, Total body surface area; NA, unavailable; T, experimental group; C, control group; M, male; F: female; CON, control group with routine care; STAIC, The State Trait Anxiety Index for Children; WBFRS, Wong/Baker Faces Rating Scale; NAPI, The Nursing Assessment of Pain Index; VAS, Visual analog scale; FT, The Fear Thermometer; OBSD‑R, Observational Scale of Behavioral Distress‑Revised.

Outcome: ①=Pain ②=Anxiety ③=hear rate ④= Respiratory rate.
